# Development and optimization of nanoparticles loaded with erucin, a dietary isothiocyanate isolated from *Eruca sativa:* Antioxidant and antiproliferative activities in ehrlich-ascites carcinoma cell line

**DOI:** 10.3389/fphar.2022.1080977

**Published:** 2023-01-25

**Authors:** Sharabjit Singh, Gurdeep Singh, Shivani Attri, Prabhjot Kaur, Farhana Rashid, Neena Bedi, Shafiul Haque, Essam M. Janahi, Saroj Arora

**Affiliations:** ^1^ Department of Botanical and Environmental Sciences, Guru Nanak Dev University, Amritsar, India; ^2^ Department of Pharmaceutical Sciences, Guru Nanak Dev University, Amritsar, India; ^3^ Research and Scientific Studies Unit, College of Nursing and Allied Health Sciences, Jazan University, Janzan, Saudi Arabia; ^4^ Centre of Medical and Bio-Allied Health Sciences Research, Ajman University, Ajman, United Arab Emirates; ^5^ Independent Researcher, Al Janabiyah, Bahrain

**Keywords:** erucin, central composite design, cubosomes, colon cancer, ehrlich ascites carcinoma, antioxidant assay

## Abstract

The study on Erucin (ER) has gained interest of nutraceutical and pharmaceutical industries because of its anti-cancer properties. Erucin is an isothiocyanate obtained from the seeds of *Eruca sativa* which possess certain drawbacks such as poor aqueous solubility and bioavailability. Therefore, the present study aimed at developing ER-cubosomes (CUB) by solvent evaporation technique followed by applying Central Composite Design to optimize ER loaded cubosomes. For this purpose, independent variables selected were Monoolein (MO) as lipid and Pluronic-84 (P-84) as a stabilizer whereas dependent variables were particle size, percentage of ER loading and percentage of its entrapment efficiency. The cubosomal nanocarriers exhibited particle size in the range of 26 nm, entrapment efficiency of 99.12 ± 0.04% and drug loading of 3.96 ± 0.0001%. Furthermore, to investigate the antioxidant potential, we checked the effect of ER and ER-CUB by DNA nicking assay, DDPH assay and Phosphomolybdate assay, and results showed significant improvement in antioxidant potential for ER-CUB than ER. Similarly, ER-CUB showed enhanced anticancer activity with a marked reduction in IC50 value than ER in MTT assay. These results suggested that ER-CUB produced notable escalation in antioxidant potential and enhanced anticancer activity than ER.

## Introduction

Plant based drugs are widely consumed worldwide for the treatment of various ailments such as cancer, heart disease, skin disease, diabetes *etc.* (Awadelkareem et al., 2022a; Kushwaha et al., 2020; Ambrocone and Tang, 2009). The plants of Cruciferae family are worthy source of active therapeutic constituents which show potential activities against cancer treatment (Kristal, 2002; Lampe and Peterson, 2002; Giovannucci et al., 2003; Lynn et al., 2006; Thomson et al., 2007; Munday et al., 2008; Tang et al., 2008; Traka et al., 2008). At present, the putative role of cruciferous vegetables on cancer chemoprevention is related to the bioactivity of the glucosinolate (GLS) hydrolysis products, namely isothiocyanates (ITCs), which are suggested to protect against cancer of colon, prostate, breast and lungs (Hecht, 2000; Bonnesen et al., 2001; Bianchini and Vainio, 2004; Munday et al., 2006; Zhang et al., 2006; Zhao et al., 2007; Hayes et al., 2008; Nakamura, 2009; Wu et al., 2009; Yang et al., 2009). Among the cruciferous vegetables, Rocket salad is commonly used as food and is rich in erucin (4-(methylthiol) butyl isothiocyanate), a compound structurally similar to sulforaphane (Awadelkareem et al., 2022b). Erucin (ER) is a reduced analogue of SF and is generated by enzymatic hydrolysis of glucoerucin, a glucosinolate found in high concentrations in rocket salads. Sulforaphane (SF) has been reported to inhibit tumour angiogenesis, metastasis, and cell migration effectively (Barillari et al., 2005; Gupta et al., 2010). The earlier pharmacokinetic/dynamic studies conducted in our laboratory indicated that the 4-(methylthio) butyl isothiocyanate is absorbed maximally in jejunum part of intestine (Kaur et al., 2022). Taking lead from these studies and coupled with the above said fact, the present study was planned to explore its anticancer activity on colon. Colon cancer is a significant global issue and the second largest cause of mortality after lung cancer. It is caused by accumulation of genetic and epigenetic alterations in colon epithelial cells (Krishnaiah et al., 2003; Lamprecht et al., 2005; He et al., 2008; Sun et al., 2008; Masloub et al., 2016; Wang et al., 2018). Furthermore, the bioavailability of anticancer medications can be improved by using nano-drug delivery system to achieve maximum therapeutics effect at lower concentration, and reduced adverse effects. The encapsulation of ER in nanoparticles will not only prevent systemic adverse effects, but also provide effective and safe colon cancer therapy at a lower dose (Shutava et al., 2009). Advanced anticancer drug delivery systems such as anticancer nanoparticles frequently employ biodegradable polymeric nanoparticles to achieve drug-controlled release are available commercially (Champagne and Fustier, 2007; Ha et al., 2008; Wang et al., 2008; Do et al., 2010; Shieh et al., 2010; Wang and He, 2010; Wu et al., 2010; Danafar et al., 2017). Various techniques for encapsulating ER have recently been developed in order to increase its therapeutic efficacy (Sinha et al., 2004; Wu et al., 2014). Cubosomes (CUB a novel type of lipid-based nanoparticles) are nanostructured liquid crystalline biocompatible carriers formed of amphiphilic lipids i.e. molecules with hydrophobic and hydrophilic characters like surfactants, polymers, and polar lipids in certain proportions (Liu et al., 2020). Due to their unusual physicochemical features, particularly their great capacity to encapsulate a wide range of active molecules from the hydrophilic, hydrophobic, and amphiphilic classes, cubosomes have attracted a lot of attention in recent years. Moreover, their propensity to self-assemble under particular conditions make these amphiphilic molecules play a significant role in drug delivery system (Ou et al., 2018; Rajendran et al., 2022). Some of the unique advantages of this class of nanocarriers include protecting the loaded actives against chemical or physiological degradation under *in vivo* conditions, and reduced adverse effects observed during drug administration (Leaf et al., 2022). The present study is focused on the development and optimization of cubosomes loaded with erucin isolated from seeds of *Eruca sativa* and evaluating their effectiveness as antioxidant and antiproliferative agents against colon cancer (Angelova et al., 2013).

### Chemical and regents

Monoolein (MO) was obtained from TCI Chemicals Pvt. Ltd. (Mumbai, India), Pluronic-84 (PE/P84) was purchased from Sigma Aldrich (Mumbai, India). Acetonitrile (ACN) and Dimethylsulfoxide (DMSO) (HPLC-grade) solvents were obtained from Finar Chemicals Pvt. Ltd. (Mumbai, India). Colon cancer cell line Ehrlich-Ascites Carcinoma (EAC) was acquired from the National Centre for Cell Sciences (NCCS, Pune, India). The chemicals used for cell line studies were obtained from HiMedia (India). None of the other analytical-grade chemicals were further purified before usage.

### Isolation and characterization of erucin

**FIGURE 1:**
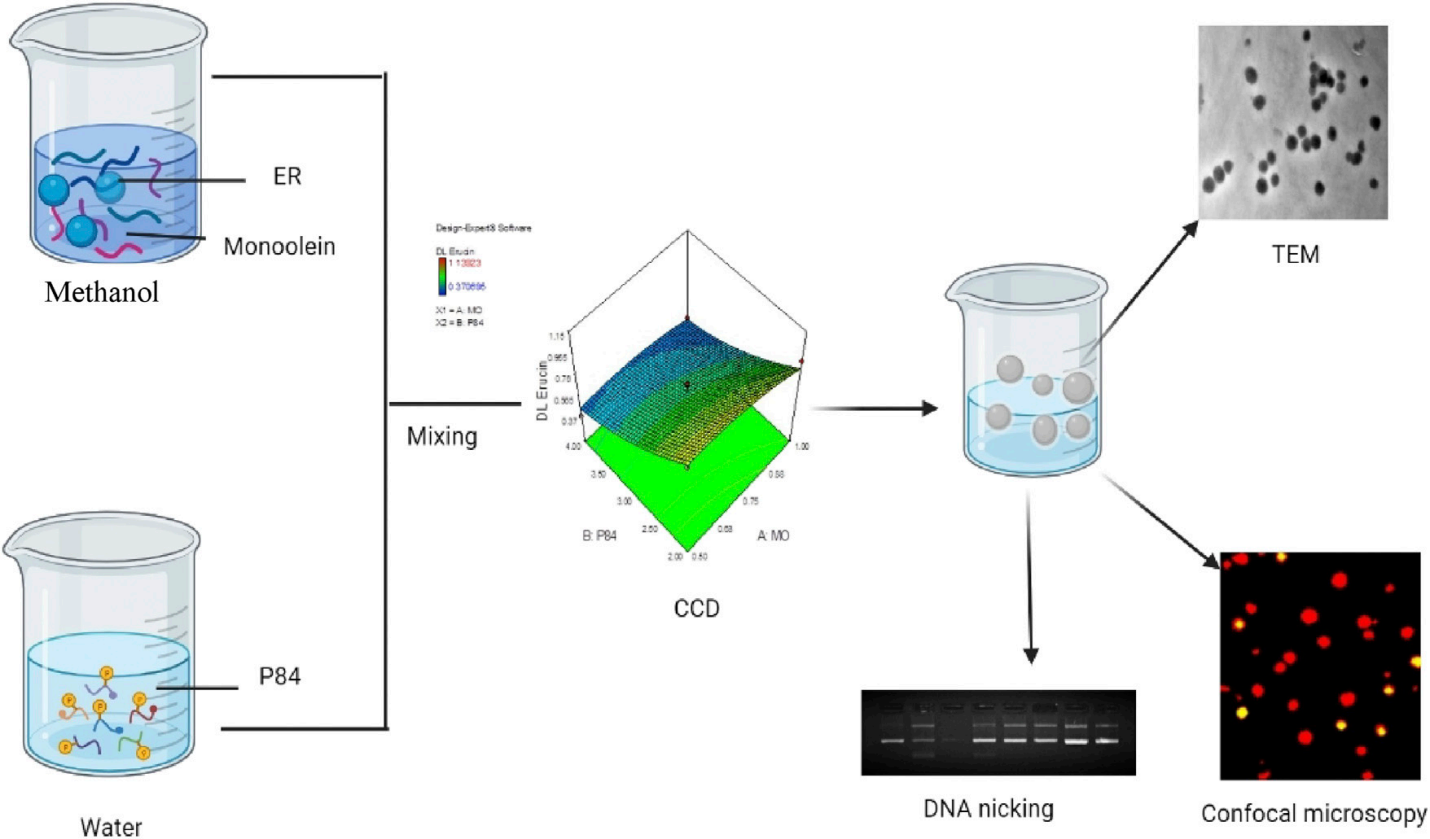
Schematic representation of preparation of ER loaded Cubosomes (ER-CUB) and *in vitro* cytotoxitity studies.

The plant material (seeds) of the *Eruca sativa* (Mill.) Thell were procured from Sri Karan Narayan College of Agriculture (SKN) Jobner, (Rajasthan, India). The identification and authentication of the plant material was done by Mr. Ram Prasad, Herbarium In-charge of Department of Botanical and Environmental Sciences (DOBES), Guru Nanak Dev University, Amritsar where the voucher specimens (7,297) were submitted. The extraction of the oil from the seeds was done by hydrodistillation method with minor modifications following the standard protocol (Figure 1, Supplementary Figures S1, S2) (Kaur et al., 2022).

### Octanol/water partition coefficient

1-octanol has a hydrophobic, long alkyl chain and a polar OH group, just like membrane lipids. Therefore, octanol-water partition coefficient best represents the behavior of a drug compound toward a membrane. The 1-octanol/water partition coefficient of the ER was measured by shake-flask method (Congliang et al., 2007). Before performing the experiments, both solvents were mutually saturated. 5 ml of the solvent-I (octanol) was added to 5 ml of the solvent-II (water) in glass flask and 100 µl of ER was added. The contents of the flask were vigorously shaken and mixture was allowed to stand in water bath kept at constant temperature of 25°C ± 1°C for at least 24 h to achieve the partitioning equilibrium. Further, the organic phase was separated from aqueous phase and the drug concentration was determined by HPLC method. All the partitioning experiments were repeated in triplicate.

### Central composite design (CCD) for cubic nanoparticles (CUB) of erucin (ER)

Two factor CCD design was applied to investigate the effect of independent variables i.e. MO (X_1_) and P84 (X_2_) on dependent variables *viz.* particle size (PS) (Y_1_), entrapment efficiency (% EE) (Y_2_) and drug loading (%DL) (Y_3_). Thirteen experiments were derived from CCD through Design Expert^®^ Software (version 7.0, Stat ease Inc., Minneapolis, MN, United States). CCD includes five replicates of the centre points, four axial and four factorial points (Varshosaz et al., 2010). Repeatability of the method used was determined by repeating centre point five times. A response surface analysis regression was employed for data analysis. The significant terms (*p* < 0.05), the least significant lack of fit, coefficient of variance, and various correlation coefficients offered by Design Expert software led to the selection of a polynomial model. The upper and lower limits of independent variables are given in Table 1.

**TABLE 1:** Central composite design of experiment and measured responses of ER-CUB.

Runs	Copolymers		Responses (mean ± SD)		
	A: MO (X_1_)	B: P84 (X_2_)	Size (nm) (Y_1_)	% EE (Y_2_)	% DL (Y_3_)
1	1.00	4.00	416.26 ± 0.92	93.29 ± 0.21	4.10 ± 0.0011
2	0.50	4.00	154.26 ± 1.18	91.65 ± 0.86	4.30 ± 0.0009
3	0.75	3.00	63.033 ± 0.80	94.23 ± 0.20	5.41 ± 0.0013
4	0.50	2.00	749.73 ± 14.4	99.59 ± 0.02	7.81 ± 0.0411
5	1.00	2.00	133.03 ± 0.83	99.12 ± 0.06	7.37 ± 0.0004
6	0.75	3.00	96.633 ± 0.36	99.61 ± 0.03	5.58 ± 0.0001
7	0.75	4.41	26.293 ± 1.90	99.12 ± 0.04	3.96 ± 0.0001
8	0.40	3.00	108.83 ± 0.55	99.34 ± 0.05	5.86 ± 0.0003
9	0.75	3.00	135.96 ± 1.25	99.09 ± 0.02	5.56 ± 0.0004
10	0.75	3.00	94.880 ± 0.39	99.37 ± 0.06	5.57 ± 0.0002
11	0.75	1.59	108.63 ± 0.77	99.16 ± 0.06	8.97 ± 0.0475
12	0.75	3.00	1,409.3 ± 178.7	99.39 ± 0.06	5.57 ± 0.0003
13	1.10	3.00	102.26 ± 0.58	90.14 ± 0.11	5.06 ± 0.0039

### Statistical analysis

The optimum concentration of the independent variables (viz. MO and P84) for CUB was selected on the basis of the responses obtained viz. minimum PS, higher % EE and %DL. The response surface nature was investigated for response function (Y) using the polynomial Eq. 1.

$$Y=\beta_{0\;}+\beta_{1}X_{1}+\beta_{2}X_{2}+\beta_{11\;}X_{1}^{2\;}+{\beta_{22}X}_{2}^{2}+\beta_{12}X_{1}X_{2\;}$$ (1)

Where Y is the predicted response; *β*_0_ is constant; *β*_1_ and *β*_2_ are the linear, quadratic and interaction coefficients, respectively. The significance of the differences between the independent variables was determined using the analysis of variance (ANOVA). The reduced model contained all significant independent variable effects (*p* < 0.05). Three dimensional response surface plots were composed in order to determine the interaction effect of the independent variables. For models with good fit, the *R* ^2^ should be at least 0.8.

### Verification of models

Established model was validated using a quantitative comparison between the theoretical and practical prediction by employing Student’s t*-*test at (*p* < 0.05, considered significant).

### Preparation of ER loaded cubic nanoparticles

The solvent evaporation method was used to prepare the ER loaded CUB formulation (Sung et al., 2005; Flak et al., 2020). Briefly, pre-weighed amount of MO and ER were co-dissolved in methanol and allowed to stir for 30min at 40°C using magnetic hot plate (Cole Parmer Mumbai, India). In another beaker, pre-weighed quantity of P84 was dissolved and allowed to stir for 30 min at 40°C. The above lipid and ER mixture was poured drop wise using micropipette into the P84 solution at constant stirring for 1 h at 40°C/100rpm. Furthermore, the organic solvent was completely removed using Rota evaporator (Ika, India) at 40°C for 1 h. The final formulation was stored at 4°C for further studies.

### Size, zeta potential and polydispersity index analysis

The size, zeta potential and polydispersity index (PDI) of the (Blank cubosomes, BLK-CUB), Erucin loaded cubosomes, (ER-CUB) were evaluated by dynamic light scattering (DLS) analysis using the Zetasizer Nano ZS-90 from Malvern Instruments (UK). About 2 ml of the sample was placed in disposable cuvette and injected into a folded capillary electrophoresis cell for zeta potential measurement. The diameter used to be calculated from the autocorrelation function of intensity of light scattered from the CUB formulation. DLS data was generated at 25°C with a fixed 90° light incidence angle. All measurements were replicated of three experiments (Singh et al., 2021b).

### Fourier transforms infrared spectroscopy (FTIR-ATR)

Chemical interactions were studied by FTIR-ATR spectroscopy. For FTIR-ATR analysis, the spectra of ER, MO, P84, Physical mixture, BLK-CUB and ER-CUB were verified by FTIR-ATR spectrophotometer (Anton Paar India Pvt. Ltd.) in the range of 4,000–400 cm^−1^ (Patil et al., 2019).

### Entrapment efficiency (%EE) (Y_2_) and drug loading (%DL) (Y_3_)

%EE and %DL was determined by separating the free ER from ER-CUB through filtration/centrifugation technique. The samples were diluted with ACN and centrifuged at 2000 rpm for 10 min. The un-entrapped ER presented in supernatant was quantified using HPLC (High Performance Liquid Chromatography) with photodiode array detector (HPLC-PDA) (Naxera model, Shimadzu Asia Pacific Ltd.) using C18 column (150 mm × 5 μm) at a flow rate of 0.3 ml/min with injection volume of 10 μl at detection wavelength 254 nm. The %EE and %DL of ER were calculated by using of the following equations:

$$EE\%=\frac{Weight\;of\;the\;drug\;in\;cubosomes}{Weight\;of\;the\;feeding\;drug}\times 100$$ (2)

$$DL\%=\frac{Weight\;of\;the\;drug\;in\;cubosomes}{Weight\;of\;the\;feeding\;excipients\;and\;drug\;}100$$ (3)

Where-cubosomes stands for CUB (Zhan et al., 2012).

### Transmission electron microscopy (TEM)

Using TEM (JEM-1200EX, JEOL, Tokyo, Japan), morphological examination of the ER-CUB was carried out. A drop of freshly made CUB dispersion was applied to a copper grid to prepare the samples for TEM, and they were then stained with a phosphotungstic acid solution (2% w/v). The air-dried samples were then analysed using TEM with a 200 kV accelerating voltage (Singh et al., 2021a).

### *In vitro* release studies

*In vitro* release studies for Free ER suspension and ER-CUB were performed using dialysis bag method in 0.1 N HCl (pH 1.2), phosphate buffer saline (PBS) pH 6.8 and PBS pH 7.4. The optimized test samples (equivalent to 8 mg of ER) were filled in dialysis bag (MW cut-off of 12,000Da, HiMedia Pvt. Ltd.), immersed in 100 ml of release media, stirred at 100 rpm and maintained at 37°C ± 0.5°C. At predetermined time intervals, 1 ml aliquot of release medium was withdrawn and replenished with equal volume of the same for maintaining sink conditions. The amount of ER released was determined using HPLC analysis. The *in vitro* release of all test samples was conducted in triplicate (*n* = 3) and compared with free ER suspension (Mahajan et al., 2021).

### Cytotoxicity evaluation using MTT assay (3-(4,5-dimethylthiazol-2-yl)-2,5 diphenyl tetrazolium bromide) assay

The cytotoxic potential of the ER and ER-CUB was checked out by using MTT assay (Soltan-Dallal et al., 2017). EAC cells were sowed in 96-well plates and treated with 100 μl of fresh medium having pure ER and ER-CUB. After incubation of EAC cells for a definite period, 3-(4,5-dimethylthiazol- 2-yl)-2,5-diphenyltetrazolium bromide (MTT) solution (5 mg/ml) freshly prepared in PBS was added to the cell content solution. The plates were incubated for further 3 h, followed by the addition of Dimethylsulfoxide (DMSO) to fade formazan crystals. The absorbance was recorded at 570 nm using ELISA reader (Biotech Synergy HT). The percentage cytotoxicity was calculated as follows:

$$Inhibition\;\left(\%\right)=\left(Ac-\frac{As}{Ac}\right)*100$$ (4)

whereas Ac = control absorption and As = sample absorption.

### Cellular uptake studies

The cellular uptake studies were performed using acridine-orange (AO) and ethidium-bromide (EtBr) (AO/EtBr) staining with Fluorescence microscopy. The mechanism of cell death and alterations in nuclei during apoptosis was analyzed in EAC cells using AO/EtBr staining with method suggested by (Attri et al., 2022). For 24 h, EAC cells (4×10^5^/well) were treated with ER, BLK-CUB and ER-CUB. Suspended and attached cells were pooled together and centrifuged at 2,500 rpm for 5 min to form a pellet. Cell pellet was further suspended in 1× PBS (100 μl). Ultimately, cells were incubated for 5 min after mixing with 5 μl mixture of AO/EtBr (60 μg/ml (acridine orange)/100 μg/ml (ethidium bromide)). On a microscopic slide, 25 μl of stained cell mixture was poured, covered with a coverslip and immediately seen under a fluorescence microscope after making slides. Fluorescence microscopy was performed to investigate the cellular cytotoxicity of ER and ER-CUB preparations using 4,6-diamidino-2-phenylindole staining (DAPI, 10 μg/ml). The 24-well plates containing sterilized coverslips cultured with EAC cells. The cultured cells were treated with different preparations and further incubated for 24 h. Following incubation, the cover slips were placed on glass slides, and images were captured using a fluorescence microscope (Nikon Corporation, Japan) (Nankali et al., 2020).

### *In-vitro* antioxidant analysis

Most of the diseases occurring world-wide are the result of a series of uncontrolled reactions initiated by a number of reactive species (ROS). To counteract the effect of these reactive species involved in different diseases, ER must be able to stabilize these and thus act as a successful antioxidant (El-Gayar et al., 2022).

### Hydroxyl radicals scavenging activity

This activity was performed by DNA nicking assay to determine the ability of ER and ER-CUB to protect super coiled pBR322 DNA from devastating effects of Fenton’s reagent which generates the hydroxyl radicals. DNA nicking assay was performed as described by Kumar et al., 2020 (Kumar et al., 2020). In this assay, 0.5 μl of plasmid DNA was mixed with 10 μl of Fenton’s reagent (30 mM H_2_O, 50 μM ascorbic acid, and 80 μM FeCl_3_) followed by the addition of 10 μl of various dilution of ER and ER-CUB. The final volume of the mixture was brought up to 22 μl using distilled water. The reaction mixture was incubated for 30 min at 37°C and the DNA was loaded on 1% agarose gel (prepared by dissolving 0.5 g of agarose in 50 ml of 1X TBE buffer followed by ethidium bromide staining). DNA was analysed by observing the gel under UV radiation. Rutin was taken as a standard. Densitometry analysis was done by using BIO-RAD software.

### DPPH (2-2-diphenyl-1-picrylhydrazyl) radical scavenging assay

The free radical scavenging activity of ER, BLK-CUB and ER-CUB was measured in terms of hydrogen donating or radical scavenging ability using stable DPPH radicals (Blois, 1958). 200 ml of DPPH solution (0.1 mM) in methanol was mixed with 20 μg of ER and ER-CUB at different concentrations. Furthermore, after 30 min the absorbance was measured at 517 nm. Gallic acid was used as the reference compound. Lower absorbance of the reaction mixture indicated higher free radical scavenging activity (Zhang et al., 1992; Gustafsson et al., 1996; Spicer et al., 2001; Esposito et al., 2003; Barauskas et al., 2005; Wörle et al., 2007; Peng et al., 2010; Soni et al., 2017; Golombek et al., 2018; Paudel et al., 2018). Radical scavenging activity was expressed as the inhibition percentage of free radical by the extract/fraction and was calculated using the following formula: % Inhibition = [(A_C_-A_E_)/A_C_× (100)] where, A_C_ is the absorbance of the control, A_E_ is the absorbance in the presence of sample. All tests were performed in triplicates and plotted with the mean values.

## Reducing power assays

### Ferric ion reducing antioxidant power (FRAP) assay

The iron (III) reductive capacity was determined using the method given by Oyaizu (1986), Chitra et al. (2017). 1 ml each of Pure ER in methanol, BLK-CUB and ER-CUB of different concentrations was mixed with 2.5 ml of phosphate buffer and 2.5 ml of 1% potassium ferricyanide [K_3_Fe(CN)_6_]. The mixture was incubated at 50°C for 20 min. At the end of the incubation, 2.5 ml of 10% trichloroacetic acid was added. The supernatant (2.5 ml) was mixed with 2.5 ml of distilled water and 500 μl of freshly prepared ferric chloride (1%) and absorbance was measured at 700 nm. Increased absorbance of the reaction mixture indicated increased reducing power. Ascorbic acid was used as a positive control. Reducing ability is expressed as the percentage reduction of iron (III) by the samples. % Reduction = [1- (A_Max_-A_E_/A_Max_)] ∗ 100, Where, A_Max_ = maximum reading of standard after deducting control reading. A_E_ = reading of the samples after deducting that of control. All the tests were performed in triplicates and plotted with the mean values. The increase in the reducing ability of the sample was due to an increase in the absorbance of the reaction mixture, and the results obtained were compared with standard antioxidant ascorbic acid.

### Molybdate ion reduction assay

The capacity of ER, BLK-CUB and ER-CUB to reduce molybdate ions was analyzed. An aliquot of 0.3 ml of 500 μg/ml concentration of *E. sativa* seeds was combined with 3 ml molybdenum reagent (50 ml of 0.6 M sulfuric acid, 50 ml of 28 mM sodium phosphate and 50 ml of 4 mM ammonium molybdate). The reaction mixture was incubated in a water bath at 95°C for 90 min. After cooling to room temperature, the absorbance was measured at 695 nm. The gallic acid was taken as standard and standard curve was obtained using 40–200 μg/ml concentrations. The regression equation obtained for gallic acid was: y = 0.0028 x + 0.0172 (*R* ^2^ = 0.9966). Here, y = absorbance obtained at 695 nm and x = concentration of gallic acid used. The total antioxidant activity of the ER, BLK-CUB and ER-CUB was expressed as mg gallic acid equivalents (GAE)/µl of ER, BLK-CUB and ER-CUB.

### Stability study

In order to access the stability of optimized BLK-CUB and ER-CUB, all the samples were stored in stability chamber maintained at 30°C ± 2 °C/65% RH and refrigerated conditions (4°C ± 2°C) for approximately 3 months. All test samples were evaluated at different time intervals for Particle size (PS), PDI, %EE and %DL. All the measurements were taken in triplicate.

## Results

### Octanol/water partition coefficient

**FIGURE 2:**
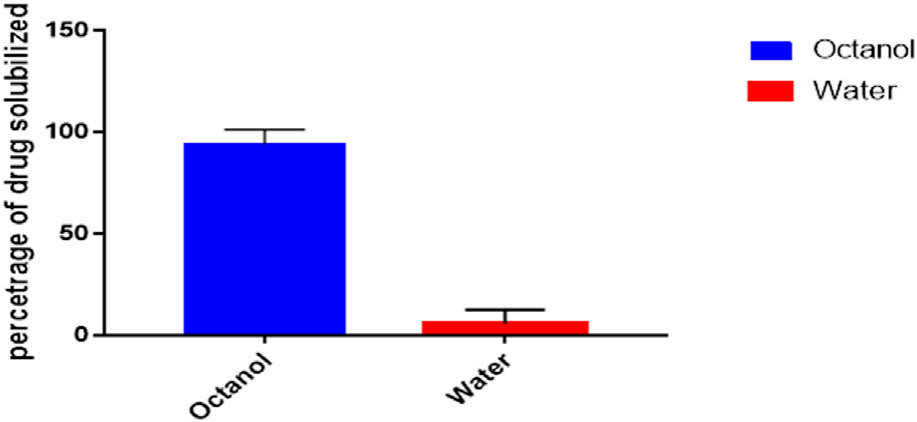
Partition co-efficient of ER using Octanol/water.

Shake-flask method was used to determine the *n*-octanol/water partition coefficients of ER and the behaviour of compound towards the mixture was observed (Figure 2) (Angelova et al., 2013). The 1-octanol/water partition coefficient (*K*OW) of ER is defined by the ratio of the compound concentration in 1-octanol phase to its concentration in water at a defined temperature which in logarithmic form is expressed as

$$\log KOW=\log\left(CO/CW\right)$$ (5)

Where *C*O and *C*W represent molar concentrations of the partitioned drugs in 1-octanol and aqueous phase at equilibrium, respectively. The molar concentration of the drugs in water (*C*W) was calculated by

$$CW=CO\left(i\right)–CO\left(f\right)$$ (6)

Where *C*O(i) and *C*O(f) are the molar concentrations of the drug in 1-octanol phase before and after the shaking, respectively.

### Design of experiment optimization formulation of CUB-ER

**FIGURE 3:**
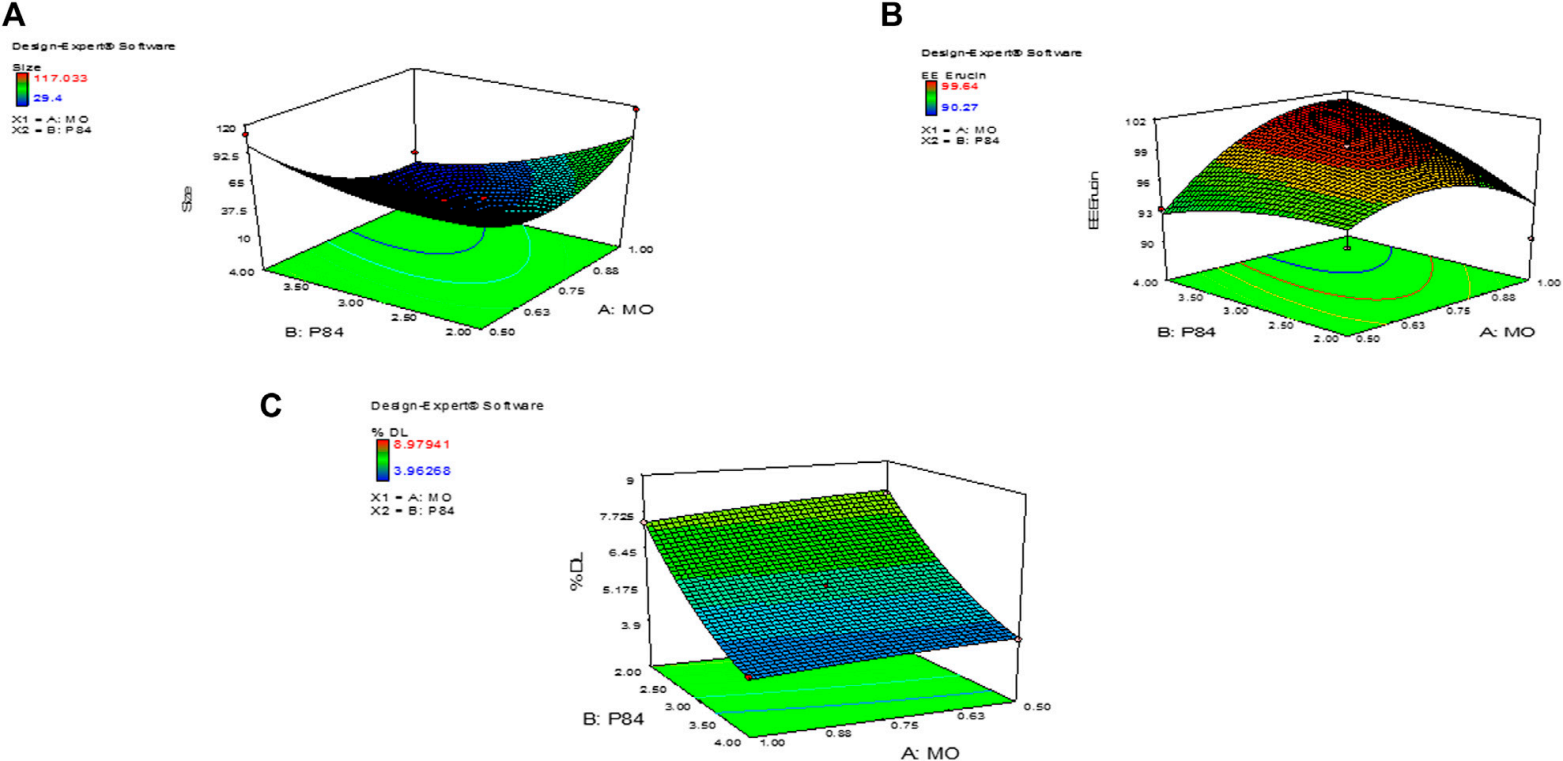
3D images of Central composite design of experiment showing **(A)** Size of ER-CUB **(B)** Entrapment efficiency **(C)** Drug loading of ER.

The variation in independent variables of formulation was predicted using response surface methodology as these responses depend on the composition of cubosomes. Statistical data analysis was used to obtain the best fit models for the independent variable of CUB. All the parameters such as regression coefficients (*R* ^2^), regression value (*p*-value) and derived equation for particle PS (nm), PDI, % EE and % DL are expressed in Table 1. If the quadratic or interaction terms including these variables were significant (*p* > 0.05), the non-significant linear terms (*p* > 0.05) were also included in the final reduced model. Significance of the quadratic polynomial models was assessed using Analysis of variance (ANOVA). All factors in the models had a large F-value and a modest *p*-value (*p* > 0.05) indicating that they had a substantial impact on the response variables. Results indicate that MO has a positive effect on the PS, PDI, %EE and %DL, while the concentration of P84 had a significant effect on PS of CUB (Nasr et al., 2021). The interaction between the variables in the design was further elucidated by the 3D response surface plots of the combination of MO and P84 (Figure 3).

### Size, zeta potential (ZP) and polydispersity index (PDI) analysis

The results of particle size analysis of the prepared Cubosomes. Mean particle size of BLK-CUB and ER-CUB were 20.452 ± 0.89 nm and 26.293 ± 1.90 nm, respectively. Both BLK-CUB and ER-CUB dispersions displayed a narrow particle size distribution. The value obtained for particle size may be attributed to the use of a relatively high concentration of P84 as a stabilizer.

$$Y_{1\;\left(Size\;ER-CUB\right)}=33.82-16.32X_{1}-11.28X_{2}-24.66X_{1}X_{2}+24.06X_{1}^{2}+12.41X_{2}^{2}$$ (7)

### Determination of %EE and %DL of ER-CUB

The loading and entrapment effectiveness of the cubosomes is generally high in binary lipid matrices, as reported in previous findings (Soni et al., 2017; Attri et al., 2022) Entrapment efficiency and drug loading of ER in cubosomes was determined by using HPLC method. % EE and % DL of the cubosomes can be logically considered to be the primary indicators of miscibility between the drug and copolymers (Supplementary Figure S3). The copolymers exhibited a significant effect on the EE and DL of the ER-CUB, as shown in Table. 1 (runs 1–13). The highest values of EE and DL were 99.5 ± 0.01% (run 4) and 8.97 ± 0.01 (run 11), respectively, indicating miscibility of the ER with copolymers

$$Y_{2\;\left(\%\;EE\right)}=99.47+1.60X_{1}+1.70X_{2\;}+2.55X_{1}X_{2}-2.73X_{1}^{2\;}+0.87X_{2}^{2}$$ (8)

$$Y_{3\;\left(\%\;DL\right)}=0.65-0.069X_{1}-0.26X_{2\;}+0.030X_{1}X_{2}-0.53X_{1}^{2}+0.075X_{2}^{2}$$ (9)

### Fourier transforms infrared spectroscopy (FTIR-ATR)

**FIGURE 4:**
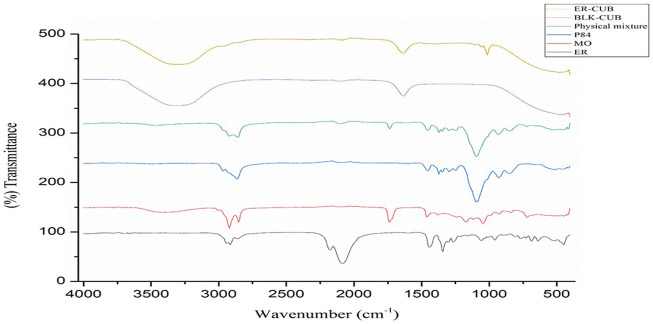
FTIR spectra of ER, MO, P84, Physical mixture, BLK-CUB and ER-CUB.

The FTIR-ATR spectra of pure ER, MO, P84, physical mixture, BLK-CUB and ER-CUB as shown in Figure 3. Pure ER showed characteristics absorption peak at 2,922.2, 1,058, and 961 cm^−1^ which is assigned to terminal C-H, C-C and CH_2_-N stretch, respectively (Figure 4). Physical mixture showed characteristics minor absorption peak at 2,922.2, 1,095.8, 931.8 cm^−1^ which is assigned to C-H, C-O symmetrical and C-O stretch, respectively similar to ER and polymers. Taken together, the overall FT-IR peaks of ER-CUB was different from that of the physical mixture and identical to BLK-CUB.

### Transmission electron microscopy analysis

**FIGURE 5:**
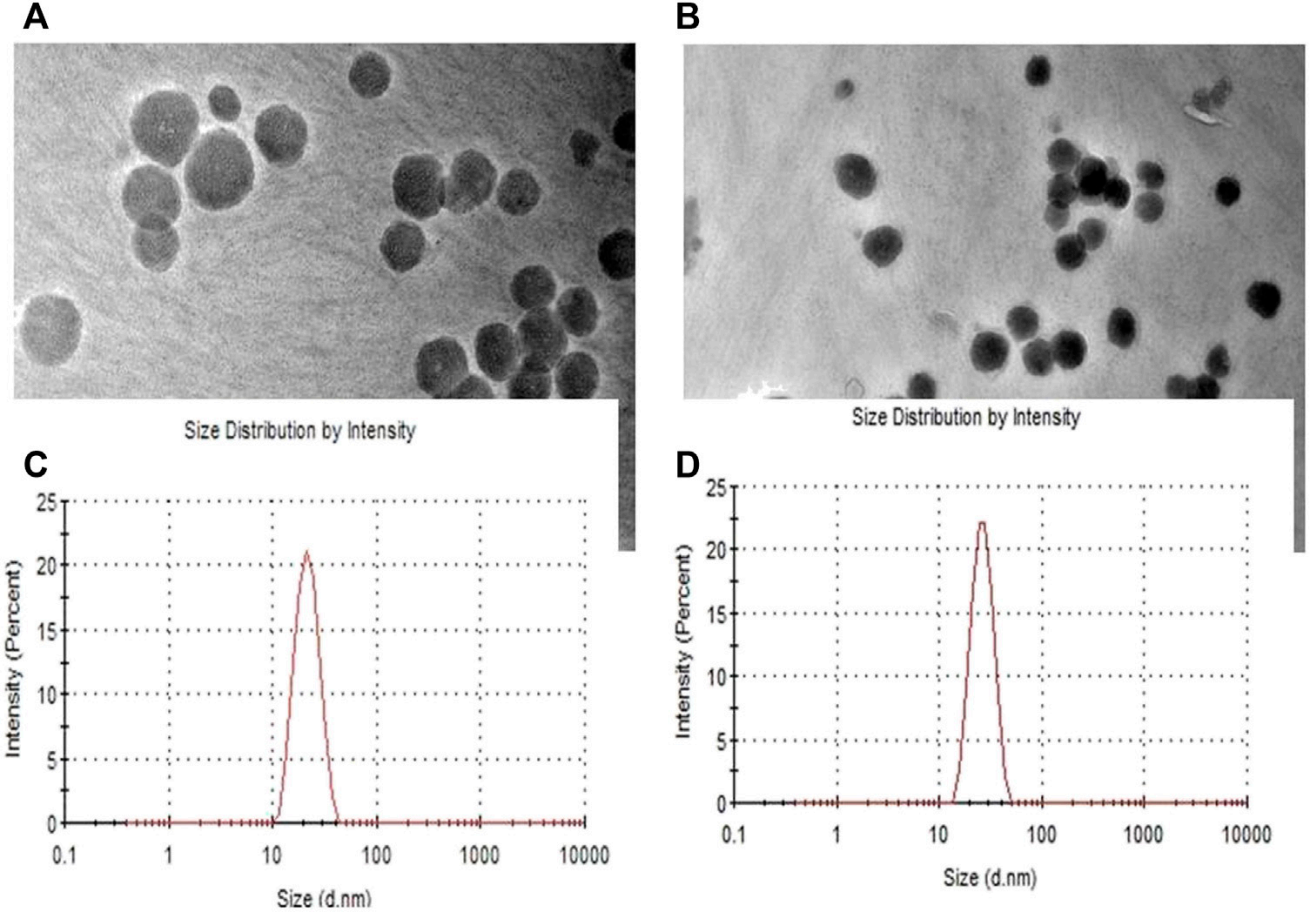
TEM images of **(A)** BLK-CUB and **(B)** ER-CUB **(C)** Size of BLK-CUB and **(D)** ER-CUB.

Morphological evaluation of BLK-CUB and ER-CUB revealed a near cuboidal shape and particle size less than 50nm, which were consistent with results obtained with DLS (Figure 5). Results suggested that encapsulation of ER in CUB did not affect the physical properties of developed formulation.

### *In vitro* ER release studies

**FIGURE 6:**
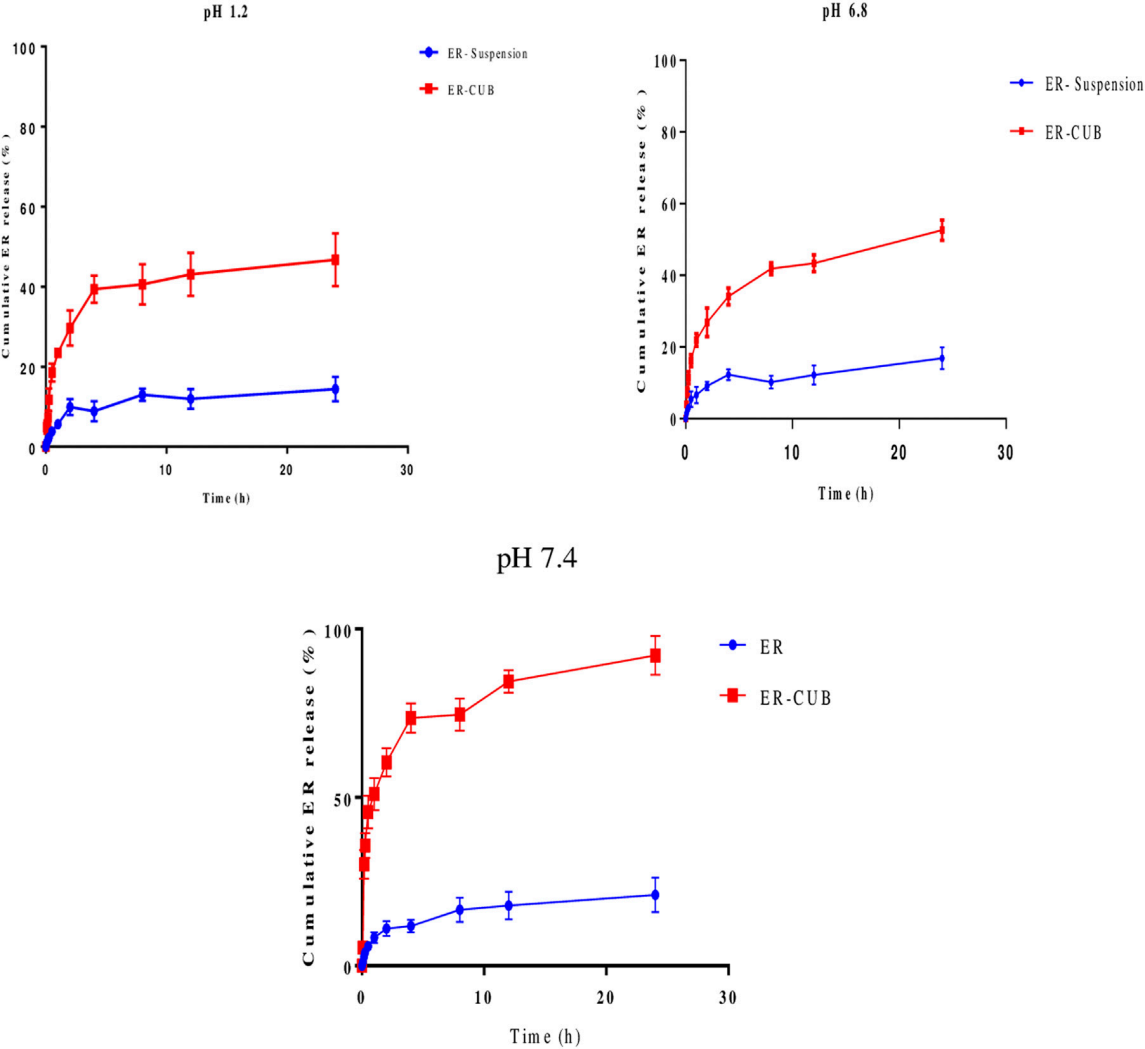
*In vitro* release of pure ER and ER-CUB using dialysis bags method. The release study was performed at 37°C at 100 rpm in 0.1N HCl, PBS (pH 6.8 and 7.4), values are mean±SD (n=3). The release ER fabricated formulation exhibited controlled release compared to pure ER (*p* < 0.05) at different time intervals.

The cumulated release profile of ER samples was analyzed, in order to determine the appropriate pattern of release with varying pH condition. Pure ER showed limited release at different pH condition as shown in Figure 6. A controlled release pattern was observed from ER-CUB in different release media after initial burst release, which can be attributed to the rapid release of ER absorbed on the surface of CUB. In the present study, maximum % CDR of ER released from the ER-suspension was 50%, meanwhile the release from ER-CUB was more than 94% in 24 h.

### MTT assay

**FIGURE 7:**
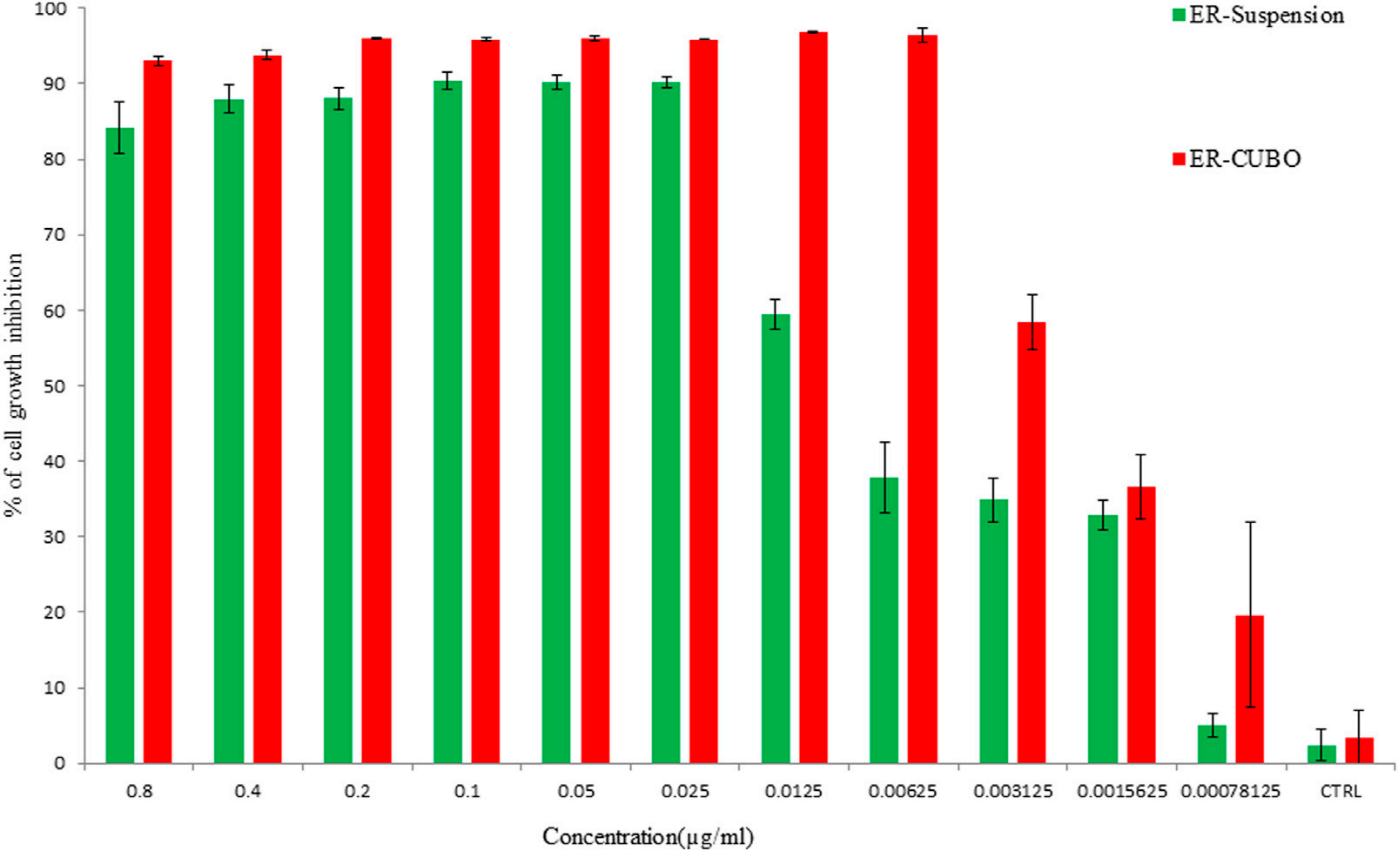
Cell viability was determined by using MTT assay. Cells were treated with varying concentrations of the ER and ER-CUB, EAC cancer cells for 24 h. Data represented as mean ± SE at the level of significance *p* ≤ 0.05.

The cell line EAC was used to check the anti-proliferative potential of the developed formulations. The anti-proliferative potential of the ER, BLK-CUB and ER-CUB was observed with varying concentrations of the ER, BLK-CUB and ER-CUB (Figure 7). Pure ER and ER-CUB exhibited the efficient growth inhibition with IC_30_ values of 0.0021 and 0.006, IC_50_ values of 0.016 and 0.062, and IC_70_ values of 0.023 and 0.084 (µl/ml), respectively (Supplementary Figure S5).

### Cellular uptake using fluorescent microscopy

**FIGURE 8:**
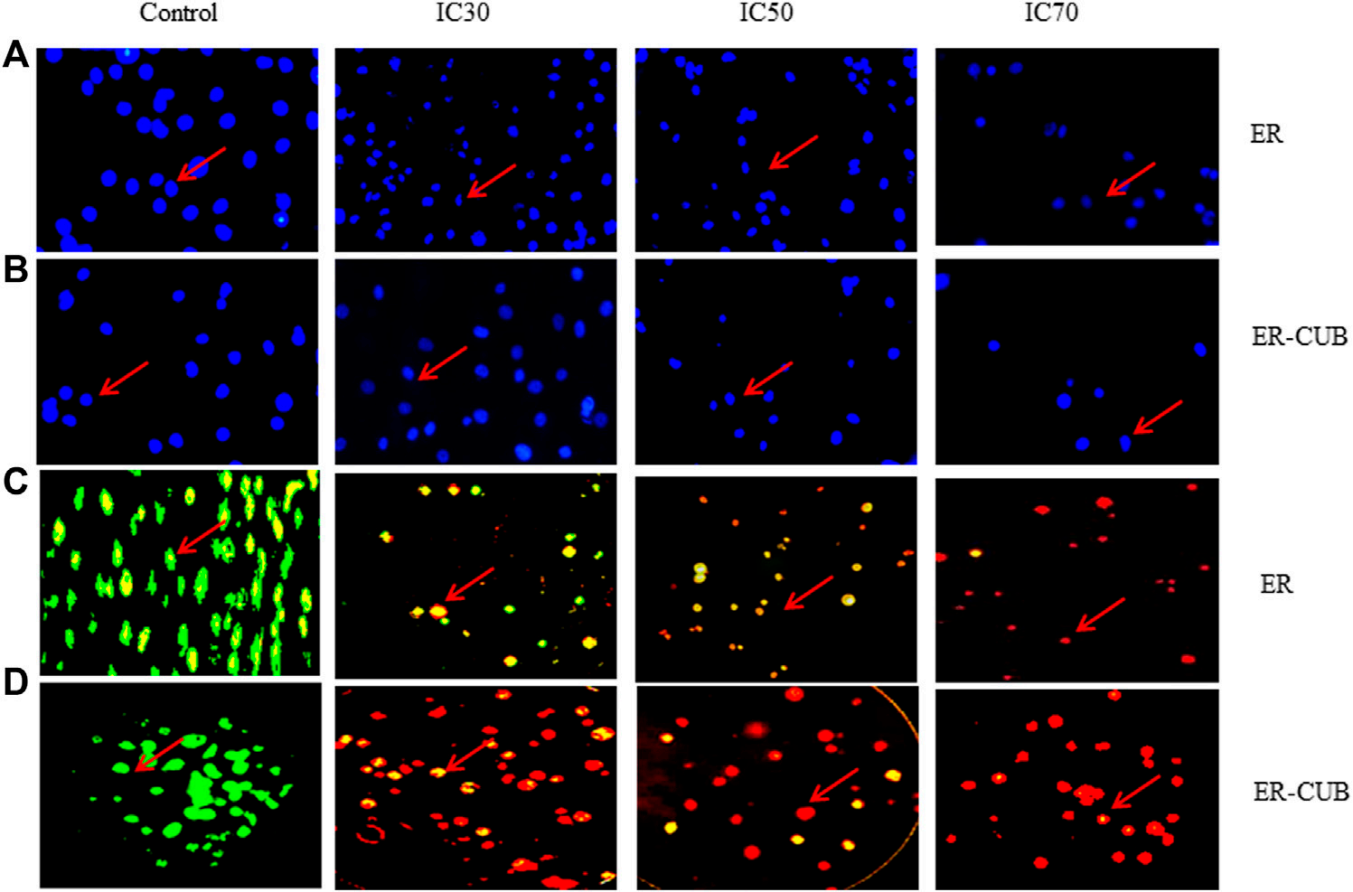
*In vitro* cytotoxicity of ER, and ER-CUB in EAC cancer cells after 24 h of incubation by MTT assay. Values are mean ± SD (*n* = 3), *p < 0.05, **p < 0.01 *versus* ER treatment at same dose. EAC cancer cells stained with Hoechst IC30, IC50 and IC70 images of acridine orange and ethidium bromide staining of EAC cancer cell line.

The fluorescence microscope observation of cells was done by staining cells with AO/EtBr as well as with Hoechst 33,342 (DAPI) stains. Treatment with 0.006 and 0.023 (µg/ml) concentrations of the ER and ER-CUB in AO/EtBr staining revealed a concentration-dependent enhancement in apoptosis using AO/EtBr staining comparative to control (Figure 8). The green staining showed control cells (viable cells), when cells given treatment with IC_50_ conc. 50% of the cells underwent apoptosis. DNA binding Hoechst 33,342 is a fluorescent stain that easily penetrates the cells and intercalates in the A-T region of the minor groove of DNA. After 24 h of treatment, EAC cells were examined for morphological changes under a fluorescence microscope. In this procedure, washing is given to cells with PBS and fixed with paraformaldehyde to evaluate morphological changes such as nuclear fragmentation and DNA condensation. Therefore, on treatment with the ER and ER-CUB, condensation and fragmentation of nuclei were observed, but no such modifications were analysed in control cells.

### *In vitro* antioxidant potential of ER, BLK-CUB and ER-CUB

The antioxidant potential of ER and ER-CUB was analyzed by performing Hydroxyl radical scavenging activity, DPPH (diphenyl-1-picryl hydrazyl), Reducing power assay and Molybdate ion Reduction Assay.

### Hydroxyl radical scavenging activity

**FIGURE 9:**
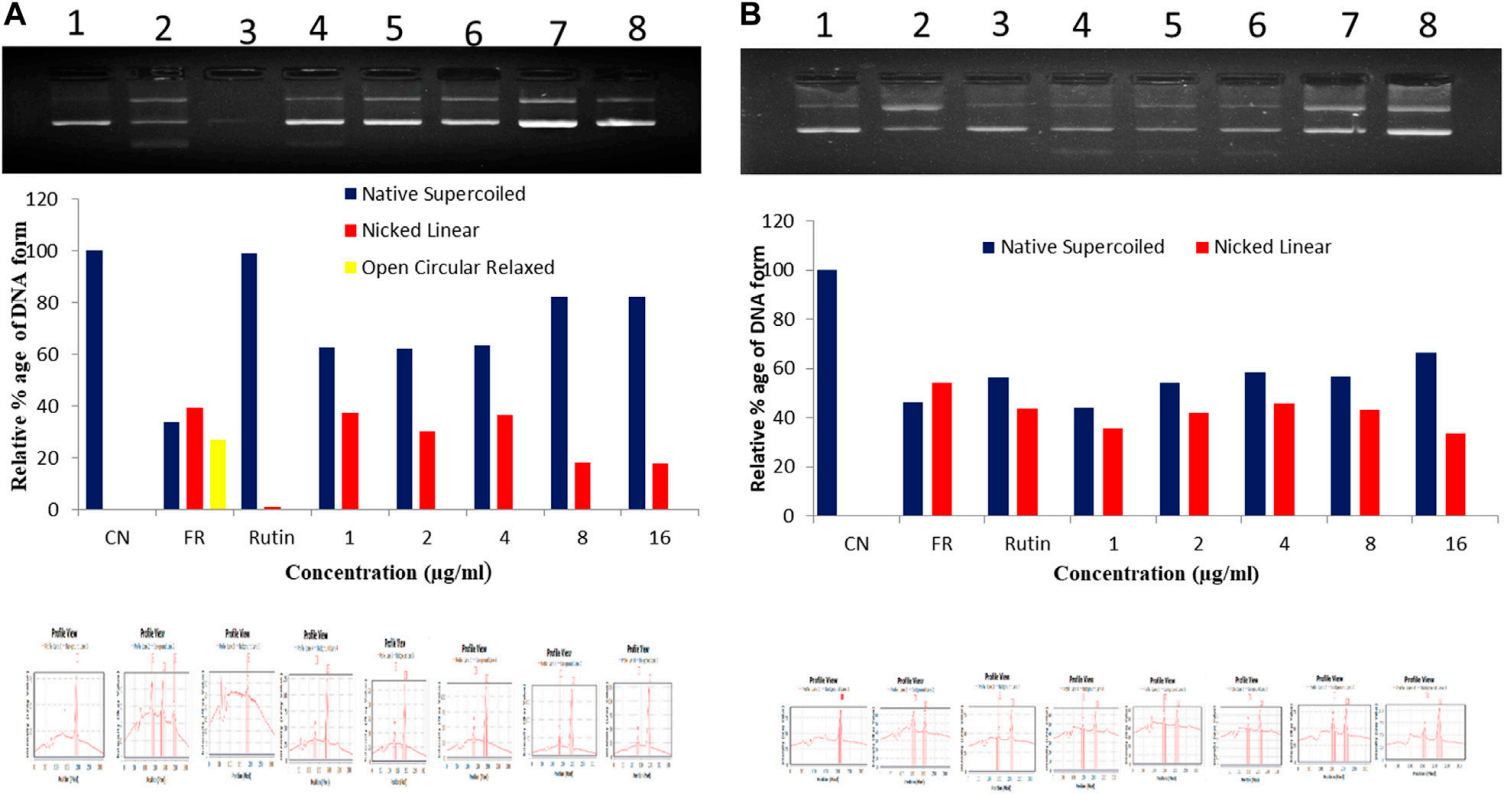
Total antioxidant capacity (TAC) of **(A)** ER-CUB and **(B)** ER (Pure) were determined using plasmid DNA on the protection of supercoiled pBR322 DNA against hydroxyl radical generated by Fenton’s reagent. CN, Negative control (pBR322 DNA + distilled water); FR, Positive control (pBR322 DNA + FR); Rutin, pBR322 DNA + FR + 100 µg/ml rutin; Conc. pBR322 DNA + FR + 1, 2, 4, 8, 16µg/ml respectively.

In hydroxyl radical scavenging activity, the ER-CUB showed the maximum protective effect towards plasmid DNA (pBR322) against hydroxyl radicals generated by Fenton’s reagent that can damage DNA strand (Form I) to nicked and open circular DNA (Form III and Form II). The addition of ER-CUB to the reaction mixture reduced the DNA strand breakage by OH radicals and preserves the native supercoiled DNA. The ER-CUB even at lower concentration exhibits a strong DNA protective effect in a dose-dependent manner (Figure 9). It preserved the native supercoiled DNA form up to 90% at the concentration of 16 μl/ml as compared to ER.

### DPPH (2-2-diphenyl-1-picrylhydrazyl) radical scavenging assay

**FIGURE 10:**
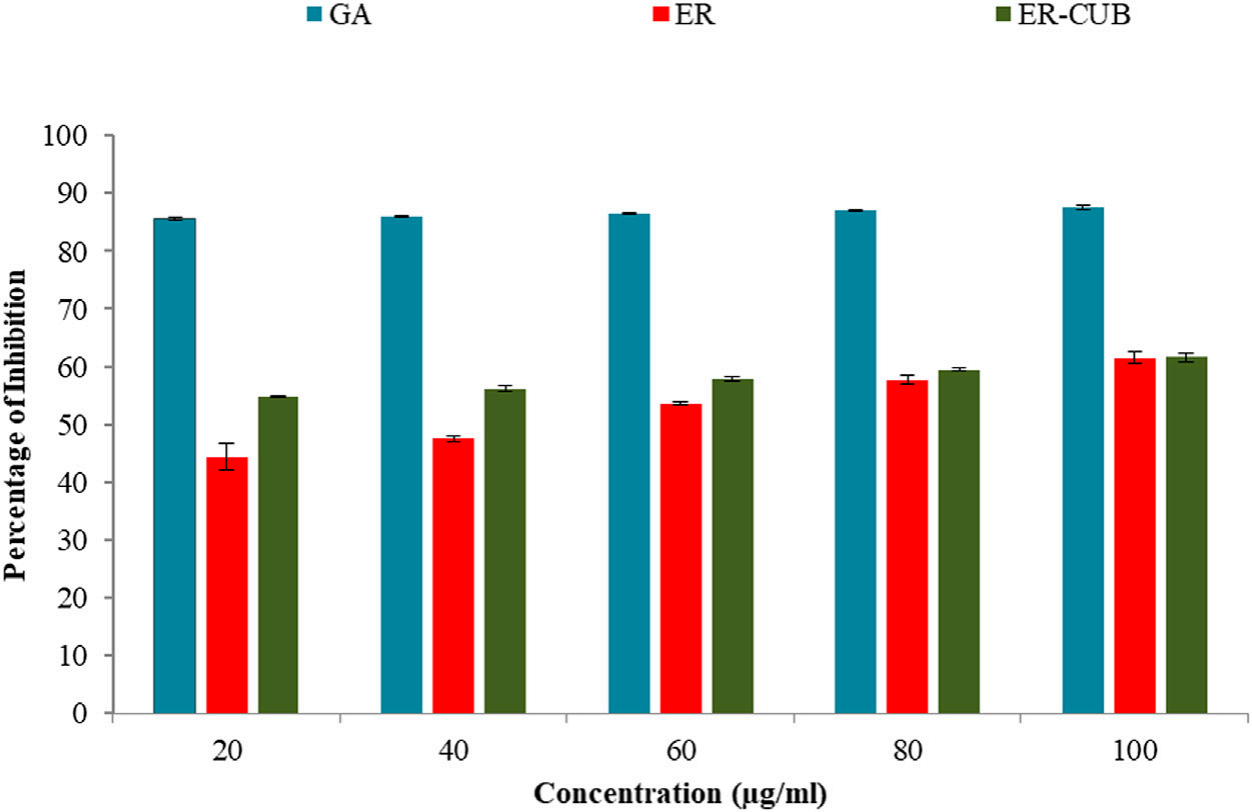
Antioxidant potential of different concentrations of ER and ER-CUB.

Among all the varied concentrations of the ER and ER-CUB, the highest concentration (100 μg/ml) showed 61.45% and 61.66% inhibition, respectively (Figure 10). Even low concentration of ER-CUB (20 μg/ml) exhibited good DPPH quenching potential of 54.85%, compared to ER which showed effect of 44.41.

### Ferric ion reducing antioxidant power (FRAP) assay

Among the different concentrations of ER and ER-CUB, the highest concentration showed potent electron-donating capacity and reduction potential with the maximum absorbance value of 0.216 and 0.088 in Ferric ion reducing antioxidant power (FRAP) assay. The reducing ability of the ER and ER-CUB was revealed by slight increase in absorbance at 700 nm, with an increase in the concentration (20–100 μg/ml).

### Molybdate ion reduction assay

The molybdate ions reduction ability of ER and ER-CUB was measured by taking gallic acid as standard. The different concentrations of gallic acid ranging from 20 to 200 μg/ml were used to obtain the standard curve. The tendency of ER and ER-CUB to reduce molybdate ions in phospho-molybdenum complex was expressed in terms of number of gallic acid equivalents (GAE) in µg/ml of ER and ER-CUB as calculated from the standard curve obtained for gallic acid. It was found that ER and ER-CUB exhibited the reduction ability of 24.47±0.52 and 38.52±0.82 μg/ml, respectively.

### Stability study

**FIGURE 11:**
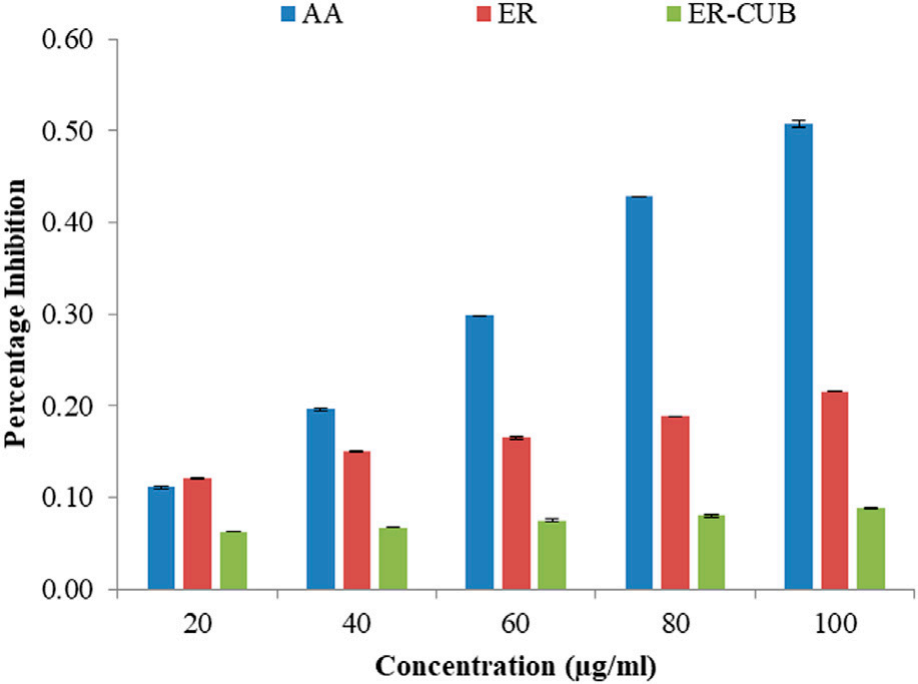
Showing the reducing potential of ER and ER-CUB in ferric reducing power assay.

ER-CUB optimized formulations were stored at 30°C ± 2°C/65% RH and refrigerated conditions (4°C ± 2°C) revealed that the particle size, PDI, and ZP showed no significant difference (*p* > 0.05) (Figure 11). ER-CUB retained ER concentration above 90% after 90 days (upto 3months) of storage under both the conditions. These results revealed that the ER encapsulated Cubic nanoparticles exhibited stability at room temperature and refrigerated conditions.

## Discussion

The purpose of present investigation was to develop an optimized formulation of ER loaded CUB using required amount of excipients to show its efficiency to enhance solubility and therapeutic potential. As a result, we have employed a methodical strategy specified by the quality by design (QbD) principles to achieve our goals efficiently and quickly. The hot emulsification technique was employed for the preparation of CUB with the objective that a similar technique could be replicated in industry with the scope of commercialization in the future. The optimized formulation was evaluated for their dynamic light scattering which showed ER-CUB loaded particle within the size range of 20–26 nm. While polydispersity of BLK-CUB and ER-CUB were 0.34 ± 0.01and 0.42 ± 0.02, respectively. The creation of mixed MO/P84 bilayers, which satirically stabilize the particles against their fusion into the cubic state, may account for the presence of primarily vesicular or porous structures with high P84 content. (Zhan et al., 2012). Although, higher P84 concentration provide smaller size particles with porous cubic structure (Singh et al., 2021a). The size of nanoparticles has a significant impact on blood circulation and bio-distribution of carrier systems. (Gustafsson et al., 1996; Worle et al., 2007; Golombek et al., 2018). The experimental design suggested that change in the proportion of MO/P84 in the binary mixture of copolymers resulted in minor changes in the EE and DL of ER in cubosome nanoparticles. This may be attributed to the higher miscibility of ER with hydrophobic copolymers. Out of the 13 runs, the lowest EE and DL were observed with an increasing proportion of hydrophilic copolymers in the binary mixture probably because of the low miscibility of ER with the higher concentration of P84 and MO (run 1). Solubilization of ER, due to its low aqueous solubility and hydrophobic nature may be favoured in the core of cubosomes nanoparticles. Additionally, cubosomes prepared with a mixture of copolymers are known to have a cube like structure. Therefore, a suitable proportion of copolymers are necessary for preparing cubosomes nanoparticles. Furthermore, ANOVA analysis indicated that the concentration of polymers had a significant (*p* < 0.001) effect on % EE and % DL. The optimization of Cubosomes for ER was formulated after evaluation of responses. Desirability, goal of the optimal formulation was to attain the maximum evaluation of responses with lower particle size, higher EE and DL. A high desirability was obtained in the CCD with higher concentration of MO and P84, respectively. The optimal copolymer proportions for ER-CUB are summarized in Table 2. To confirm the validity of the predictive models, BLK-CUB and ER-CUB were prepared using the determined optimal proportions and evaluated for particle size, % EE, and % DL. The results demonstrated that there was no significant difference (*p* < 0.05). The results indicate that characteristics ER absorption peaks were shielded by MO and P84 *via* complex formation. Moreover, there was no appearance of new peaks in ER-CUB which indicated absence of any incompatibility between the excipients. Thus, P84 and MO were found suitable for the development of cubosomes nano-formulation of ER. The results suggested that the ER entrapment in formulation does not affect the chemical and physical nature of the developed carrier system. These results suggest the controlled release behaviour of ER-CUB as compared to pure ER. The improved aqueous solubility and transition to an amorphous structure through complexation with lipid (MO) contributed to positive controlled release behaviour. The studies establish that the antitumor activity of ER is not negatively affected when the ER is incorporated into cubsomes. The relatively low cytotoxic effect of blank cubosomes as compared to ER cubosomal formulation indicates that blank cubosomes are not cytotoxic to EAC cell line. Thus, the cytotoxicity of ER-CUB is primarily due to the effect of ER present in cubosomes and the anticancer activity of ER was reported on various cancerous cell lines but effect of ER-CUB on EAC is not reported yet. ER-CUB revealed a substantial growth inhibitory outcome on tested cell line with IC_50_ value 0.0230 μl/ml. ER and ER-CUB treated cells displayed morphological shifts including viability loss, chromatin aberrations and loss of membrane integrity as exposed by using Hoescht 33,342 and AO/EtBr staining (fluorescence microscopy) (Peng et al., 2010). The protective effect of ER and ER-CUB was also tested using a plasmid (pBR322) nicking assay. Fenton’s reagent having hydroxyl radicals that can damage DNA strand (Form I) results in nicked and open circular DNA (Form II and Form III). The addition of ER and ER-CUB to the reaction mixture greatly reduces the DNA strand breakage by OH radicals and preserves the native supercoiled DNA. The ER-CUB exhibits a strong DNA protective effect in a dose-dependent manner. In DPPH assay, decolorization of DPPH occurs due to their free radicals and accepts electrons from antioxidant-rich compounds. The results of DPPH, FRAP and phosphor molybdate assay reveal the satisfactory anti-oxidant potential of ER-CUB against the free radicals. This indicated good antioxidant potential which is in concordance with the reports in literature that phytoconstiuents exhibit significant protective activity against oxidative stress (Spicer et al., 2001).

**TABLE 2:** Optimal formulation of copolymer proportion and validity of predicted responses data of ER-CUB.

Co-polymers	Optimized ER-CUB			
(A) MO (X_1_)	1:00			
(B) P84 (X_2_)	4:00			
Formulations	Responses	Predicted	Observed	Bias
ER-CUB	PS (nm)	28.6	26.2 ± 1.90	2.39
	% DL	1.00	0.48 ± 0.01	0.52
	% EE	96.8	99.1 ± 0.04	-2.30

## Conclusion

Pharmaceutical revolution of novel drugs for chronic ailments has turned many times to the plant world to identify promising bioactive phytochemicals. ER is a phytoconstituent isolated from seeds of *Eruca sativa* which was successfully incorporated into cubosomal nanoparticles. The study revealed the use of the central composite design as a data analysis approach to recognize the effect of various process variables in the optimization of key parameters. The optimized ER-CUB formulation had small particle size, high %EE and % DL. The sustained release of ER from its cubosomes can reduce the outcome related with conventional cancer therapy by decreasing the dosing frequency. The ER-CUB exhibited enhanced anti-cancer, antioxidant, *in vitro* solubility and dissolution as compared to ER alone. Furthermore, this formulation could be commercialised for treating different types of cancer and can be used in combination with commercially available anticancer drugs.

## Data Availability

The original contributions presented in the study are included in the article/Supplementary Materials, further inquiries can be directed to the corresponding authors.
