# Supplementary material for: Development and optimization of nanoparticles loaded with erucin, a dietary isothiocyanate isolated from Eruca sativa: Antioxidant and antiproliferative activities in ehrlich-ascites carcinoma cell line
Source: Front Pharmacol. 2023 Jan 25;13:1080977. doi: 10.3389/fphar.2022.1080977 (PMC9905727; doi:10.3389/fphar.2022.1080977)
Supplement: Supplementary file 1 [file Presentation1.pdf]

Development and Optimization of Nanoparticles Loaded with Erucin, a Dietary Isothiocyanate isolated from *Eruca sativa:* Antioxidant and Antiproliferative activities in Ehrlich-Ascites Carcinoma cell line

Sharabjit Singh[a], Gurdeep Singh[b], Shivani Attri[a], Prabhjot Kaur[a], Farhana Rashid[a] , Neena Bedi[b], Shafiul Haque[c], Essam M.Janahi[d*], Saroj Arora[a*I]

[a] Department of Botanical and Environmental Sciences, Guru Nanak Dev University, Amritsar 143005, India

[b] Department of Pharmaceutical Sciences, Guru Nanak Dev University, Amritsar 143005, India

[c] Research and Scientific Studies Unit, College of Nursing and Allied Health Sciences, Jazan University, Jazan-45142, Saudi Arabia

[d] Affiliation: Independent Researcher, Builiding 1848, Road 7542, Al Janabiyah 0575, Bahrain

SUPPLYMANTRY DATA

FIGURES

Figure S1 Showing a) morphology and b) seeds of the plant *Eruca sativa*

Figure S2 Extraction of ER from *Eruca sativa (seeds) using* hydrodistilation by Clevenger apparatus

Figure S3 a) High performance liquid chromatography (HPLC) Chromatogram and b) Liner curve of ER

Figure S4 Thin Layer Chromatography (TLC) of ER

Figure S5 Cytotoxicity studies of ER-CUB in EAC cell line after 24h of treatment

a) b)

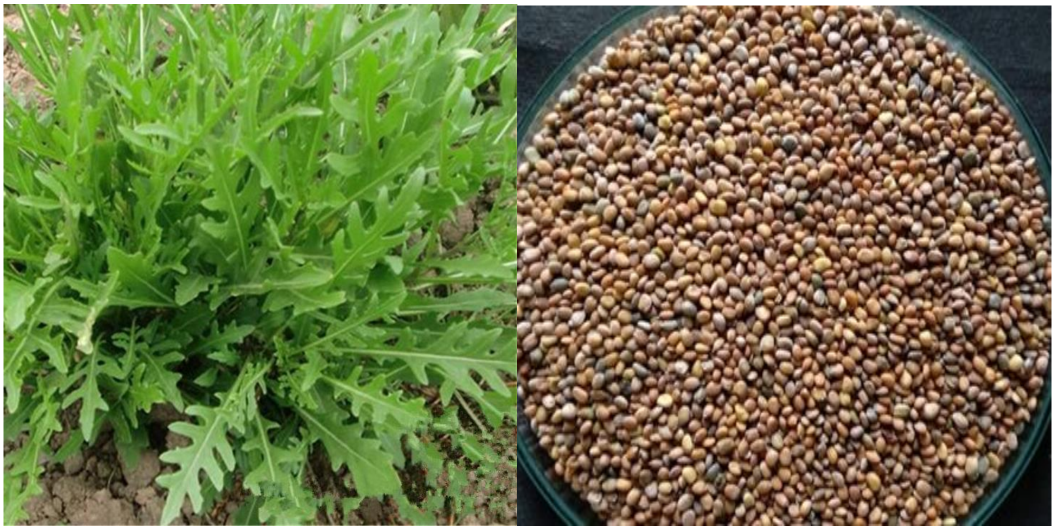

Eruca sativa (Whole plant) ***Eruca sativa (seeds)***

Fig S1 Showing a) morphology and b) seeds of the plant *Eruca sativa*

Activities: antioxidant; anticancer; antimicrobial; neuroprotective etc. Rich in ER, a glucosinolate. Hydrolyze to form a highly volatile glucosinolate hydrolytic product erucin or 4-(methylthio)butyl isothiocyanate (4-MTBITC)

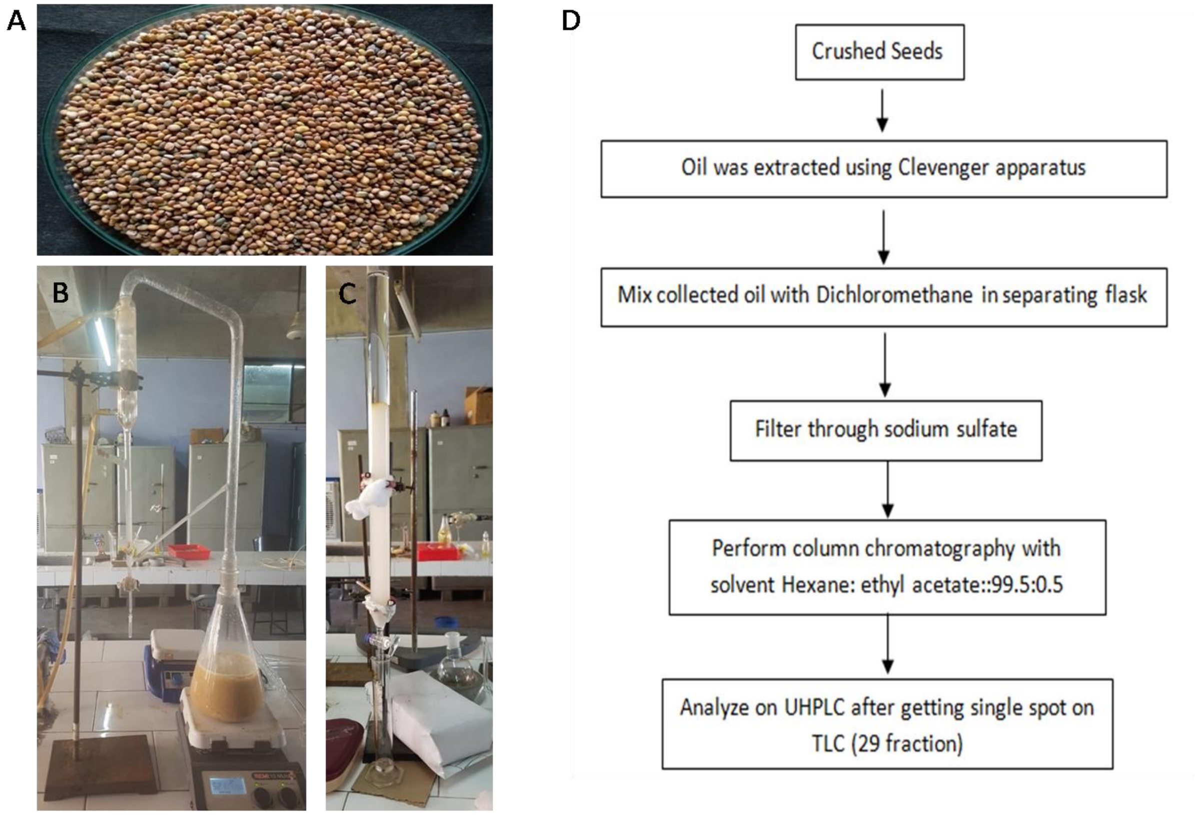

Fig. S2 Extraction of ER from *Eruca sativa (seeds) using* hydrodistilation by Clevenger apparatus

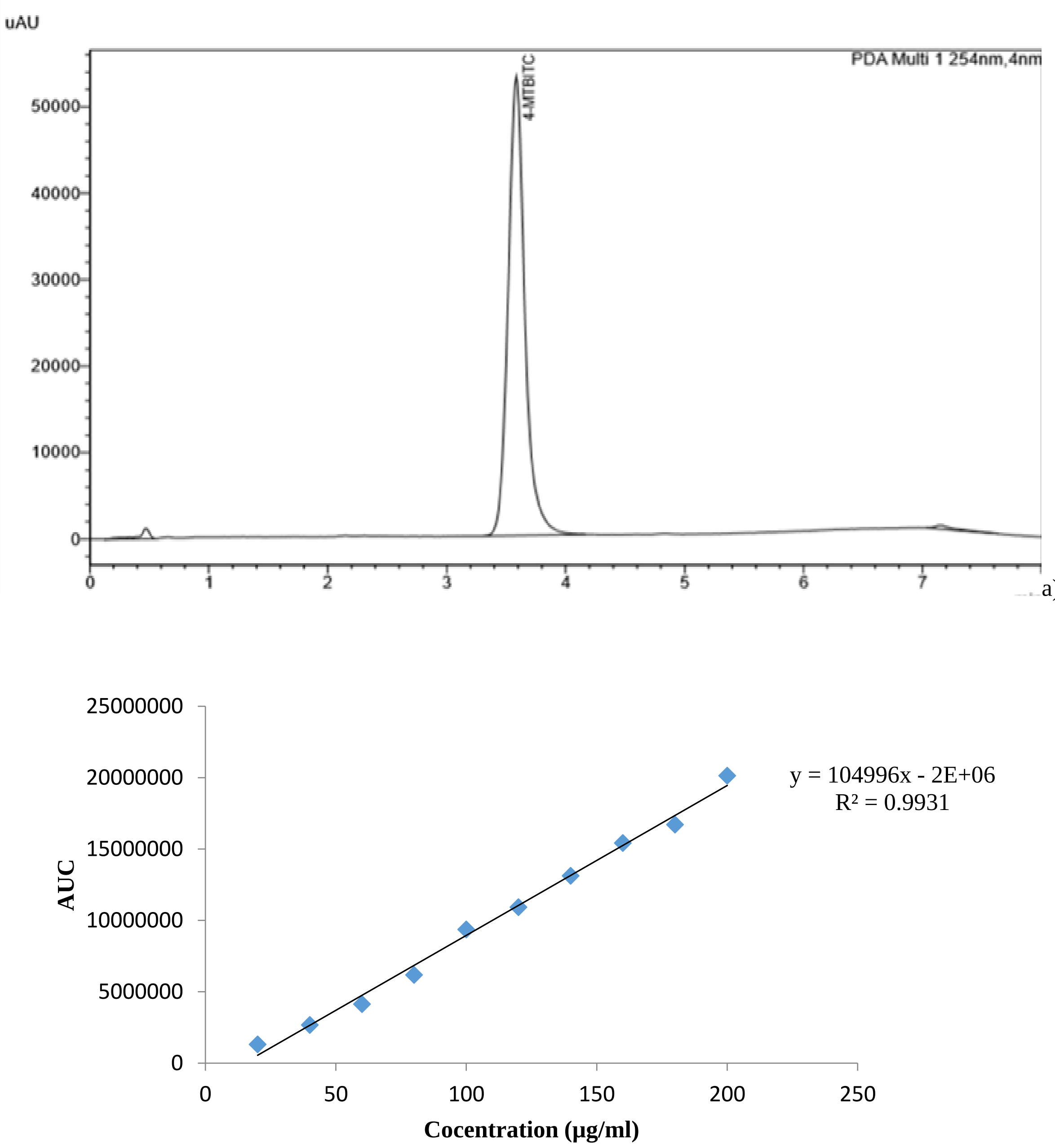

Fig. S3 a) High performance liquid chromatography (HPLC) Chromatogram and b) Liner curve of ER

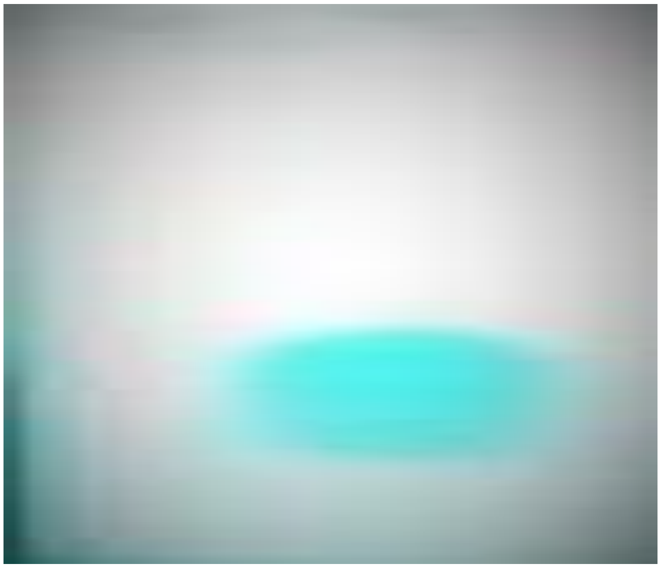

Fig.S4 Thin Layer Chromatography (TLC) of ER

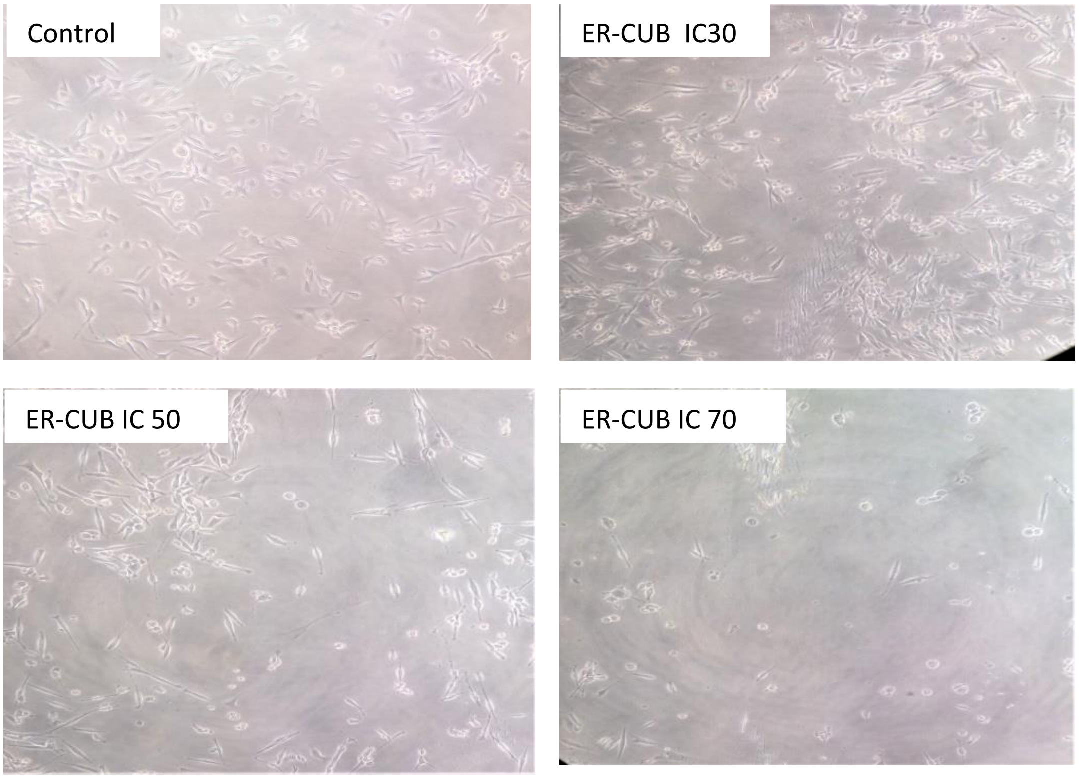

Fig. S5 Cytotoxicity studies of ER-CUB in EAC cell line after 24h of treatment
